# Physician Wellness and Burnout from Electronic Medical Record and Administrative Tasks

**DOI:** 10.5811/westjem.62945

**Published:** 2026-05-19

**Authors:** Hamza Choudry, William Adams, Jacqueline M. Dziedzic

**Affiliations:** Loyola University Chicago Stritch School of Medicine Department of Emergency Medicine, Maywood, Illinois

## Abstract

**Introduction:**

Rising physician burnout, exacerbated by the increasing complexity and time demands of electronic medical record (EMR) systems and administrative tasks, threatens physician well-being and patient care quality.

**Objective:**

To determine the impact of EMRs and administrative tasks on physician burnout in the emergency department (ED), identifying specific factors that contribute to increased burnout and decreased well-being among emergency physicians (EPs).

**Methods:**

A cross-sectional survey was sent to 53 EM attending and resident physicians at Loyola University Medical Center. We measured the following: time spent on EMR-related tasks, as well as the Maslach Burnout Inventory–Human Services Survey (MBI-HSS), which measures burnout across three domains: emotional exhaustion, depersonalization, and personal accomplishment. For each MBI-HSS domain, participants’ scores were calculated by summing the responses to domain-specific items and dividing by the number of items answered, yielding an average score that reflects the degree of burnout. Linear regression was employed to assess the relationship between each ordinal survey item and the average MBI-HSS domain score. Kruskal-Wallis tests were conducted as sensitivity analyses due to the ordinal nature of the survey items. Burnout levels were based on established thresholds to facilitate interpretation.

**Results:**

Of the 24 respondents (response rate 45.3%), 50% (n = 12) reported high emotional exhaustion, and 54.2% (n = 13) reported high depersonalization, indicating a substantial prevalence of burnout.. Weekly hours dedicated to EMR-related tasks were associated with increased emotional exhaustion (β = 0.89, SE = 0.21; p < .001) and depersonalization (β = 0.64, SE = 0.24; p = .01). Interestingly, perceived time pressure and workload related to EMR use were inversely associated with depersonalization (β = –1.19, SE = 0.34; p = .002). No significant association was found between EMR time and personal accomplishment, although 25% (n = 6) of respondents reported low scores in this domain, indicating the need to explore additional factors contributing to this aspect of burnout. 41.6% (n = 10) of subjects reported 15–20 hours per week spent using EMR, followed by 25% (n = 6) reported 10–15 hours per week and 16.6 % (n = 4) reported 5–10 hours per week and another 16.6% (n = 4) reported greater than 20 hours per week.

**Conclusion:**

This study demonstrates a significant association between time spent on EMR tasks and elevated levels of emotional exhaustion and depersonalization among ED physicians, underscoring the impact of administrative burdens on burnout. The inverse association between perceived EMR workload and depersonalization suggests that higher time pressure may paradoxically reduce feelings of detachment, potentially due to increased engagement in patient care during high-pressure situations. These findings highlight the complexity of burnout dynamics and emphasize the need for targeted interventions to optimize EMR systems and reduce administrative burdens, thereby enhancing physician well-being and job satisfaction. Further research is warranted to investigate factors influencing personal accomplishment and the complex interactions between EMR use and various dimensions of burnout in the ED setting.

